# Monkeypox: A Review of Clinical Features, Diagnosis, and Treatment

**DOI:** 10.7759/cureus.26756

**Published:** 2022-07-11

**Authors:** Asfand Yar Cheema, Oboseh J Ogedegbe, Mishaal Munir, Gabriel Alugba, Tioluwani K Ojo

**Affiliations:** 1 Medicine, Services Hospital Lahore, Lahore, PAK; 2 Internal Medicine, Lahore Medical & Dental College, Lahore, PAK; 3 Internal Medicine, Lifeway Medical Center, Abuja, NGA; 4 Medicine, Ghurki Trust and Teaching Hospital, Lahore, PAK; 5 General Medicine, Delta State University, Abraka, NGA; 6 Internal Medicine, St. Nicholas Hospital, Lagos, NGA

**Keywords:** animal-human transmission, human-human transmission, outbreaks, orthopoxvirus, viruses, endemic, mpxv, monkeypox

## Abstract

Monkeypox virus (MPXV) is an enveloped double-stranded DNA virus that results in a smallpox-like human disease. This causative organism belongs to the *Orthopoxvirus* genus. It is known to affect the neurological, respiratory, and gastrointestinal systems. The past few decades have seen endemic outbreaks of this viral infection due to the eradication of smallpox and subsequent laxity in vaccination efforts. Since it was initially diagnosed in 1970 in the Democratic Republic of Congo, it has spread to many countries worldwide, including the United States of America, becoming a disease of significant epidemiological importance. The most recent outbreak occurred in 2022. Although this viral disease is considered self-limiting, it poses serious public health concerns due to its complications and pandemic potential. This review will introduce a general overview of MPXV and describe the epidemiology, clinical features, evaluation, and treatment of monkeypox patients. It will also provide a means to raise awareness among primary and secondary healthcare providers. Furthermore, our review focuses on the most up-to-date clinical information for the effective management, prevention, and counselling of monkeypox patients worldwide.

## Introduction and background

Monkeypox is a zoonotic disease caused by the monkeypox virus (MPXV), which belongs to the *Poxviridae *family [1]. Furthermore, *Poxviridae* is subdivided into two subfamilies: *Chordopoxvirinae* and *Entomopoxvirinae*. The *Chordopoxvirinae* subfamily is known to infect vertebrates, and it is further differentiated into 18 genera, including *Avipoxvirus*, *Capripoxvirus*, *Cervidpoxvirus*, *Leporipoxvirus*, *Orthopoxvirus*, *Parapoxvirus*, *Suipoxvirus*, and *Yatapoxvirus*. The *Entomopoxvirinae* subfamily infects invertebrates, and it is grouped into four genera (*Alphaentomopoxvirus*, *Betaentomopoxvirus*, *Deltaentomopoxvirus*, and *Gammaentomopoxvirus*) [1]. There are currently 10 species in the *Orthopoxvirus* genus, including variola (smallpox) and monkeypox. MPXV is a DNA virus, but its entire life cycle occurs in the cytoplasm of the infected cells [2].

MPXV was first discovered during an outbreak among monkeys at a laboratory in Denmark in 1958 [3]. However, it was first acknowledged as a human disease in 1970, when a nine-month-old child became infected in the Democratic Republic of Congo (DRC) in an area where smallpox had been eradicated just two years earlier, in 1968 [4]. MPXV is typically found in the Congo Basin, but the other countries of Central and West Africa have also reported cases of monkeypox in humans and wildlife [5]. However, surveillance is difficult because these endemic areas lack the resources and infrastructure for epidemiological and ecological studies.

The first MPXV outbreak to occur outside of Africa was reported in the United States of America after infected African mammals were shipped from Ghana, transmitting MPXV to native American prairie dogs and spreading the disease to five states, totaling 47 reported human cases [6].

## Review

Epidemiology

The clinical entity of human monkeypox was first identified in the DRC, formerly known as Zaire, in a town called Basankusu, Equateur Province, in 1970 [4]. It was first detected in 1958 in Asian monkeys (*Macaca fascicularis*), which had been transferred from Singapore for polio vaccine research to an animal facility in Copenhagen, Denmark [3]. There are two clades of MPXV: the West African clade and the Congo Basin (Central African) clade. The Congo Basin clade, which causes a more severe disease, has a higher case fatality rate of 10.6% compared to the West African clade, which has a case fatality rate of 3.6% [7]. Many African countries are endemic to monkeypox, including Benin, Cameroon, the Central African Republic, the DRC, Gabon, Ghana, Côte d'Ivoire, Liberia, Nigeria, the Republic of the Congo, and Sierra Leone [7]. The actual public health burden of monkeypox is unknown. Between 1970 and 1980, 59 cases were reported, and after the eradication of smallpox in 1980, a five-year period of active surveillance in DRC identified 338 cases [8]. However, cases have emerged outside Africa in recent years. The first reported case outside Africa was in 2003, when Gambian giant rats imported from Ghana infected prairie dogs in the Midwestern United States, leading to 53 human cases [9]. The Sudanese outbreak of 2005 is the second monkeypox epidemic outside the Congo Basin and West African regions [2]. In 2017, 122 cases were reported in Nigeria, the first known cases diagnosed in 39 years, with studies showing both zoonotic and human-human transmission [8]. Outbreaks worldwide are usually linked to people who recently returned from endemic areas. However, World Health Organisation (WHO) reported a new outbreak in May 2022, which led to a paradigm shift with over 780 laboratory-confirmed cases as of June 2, 2022, from 27 member nations that were not endemic to MPXV and with no travel history to endemic areas [10].

Etiology

MPXV belongs to a family of double-stranded DNA viruses in the genus *Orthopoxvirus*, which also includes cowpox (CPX), variola virus (VARV), and vaccinia virus (VACV) [11]. It possesses characteristic oval or brick-shaped structures that measure 200-400 nm under an electron microscope with a lipoprotein envelope [1]. The MPXV genome is similar to other viruses in the *Orthopoxvirus* genus, characterized by a 6379-bp terminal inverted repetition, which possesses a putative telomere resolution sequence and short tandem repeats [12]. MPXV can enter the host cell by two mechanisms: first, by the fusion between ligands on the viral envelope and the host's cell plasma membrane receptors like chondroitin sulfate or heparan sulfate, after which parts of the viral envelope quickly disperse in the plasma membrane, or second, by endosomal uptake through a macropinocytosis mechanism that involves actin [11,13]. Within the cell cytoplasm, the virus then releases viral proteins and enzymatic factors that impair cellular defenses and stimulate the expression of early genes with the subsequent synthesis of early proteins, replication of DNA, and production of intermediate transcription factors [11].

As mentioned earlier, two phylogenetic clades of MPXV have been described in the literature, the Central African (Congo Basin) clade and the West African clade, with differences in genomic structures responsible for the greater virulence exhibited by the Central African clade, resulting in more severe disease with higher case fatality rates. This significant virulence is due to its ability to inhibit T cell receptor-mediated T cell activation and prevention of the production of inflammatory cytokines such as interferon-gamma (IFN-γ) and tissue necrosis factor-alpha (TNF-α) by human cells [7,14]. In addition, the Central African clade also possesses a gene that inhibits complement enzymes resulting in a crucial immune-modulating factor further contributing to its increased virulence [15]. However, studies have shown that the virulence of monkeypox does not involve the inhibition of major histocompatibility complex (MHC) expression or cellular transport of MHC molecules [15].

Transmission

Animal-human (zoonotic) transmission and human-human transmission are the two means of spreading the MPXV. Zoonotic transmission occurs through direct contact, bite or scratch from an infected animal, or consumption of an animal host, which is usually a rodent or a primate. Risk factors for zoonotic transmission of MPXV include living in forested or recently deforested areas, no smallpox vaccination, handling or eating dead bushmeat or monkeys, and sleeping on the floor (in endemic areas) [2]. Human-to-human transmission can result from close contact with respiratory secretions, skin lesions of an infected person, or contaminated objects such as clothes and beddings. However, transmission via respiratory droplet particles usually requires prolonged face-to-face contact, which puts health workers, household members, and other close contacts of active cases at greater risk [2,16]. The virus can also be transmitted vertically from a mother to a fetus, leading to congenital monkeypox. While close physical contact is a well-known risk factor for transmission, it is still unclear if monkeypox can be transmitted sexually [16].

Clinical features

Monkeypox is a self-limiting disease with symptoms lasting two to four weeks with an incubation period of 8 days (4-14 days) [17]. The initial signs and symptoms are usually non-specific, with a viral febrile prodromal phase characterized by headache, malaise, backache, fatigue, lethargy, and low-grade fever. Then 12-16 days after exposure, a vesiculopustular rash begins on the face and trunk and then spreads to other body parts, including palms and soles, in a centrifugal distribution [2,16]. Rash morphologically progresses through stages of macular, papular, vesicular, and pustular lesions [18]. The pustules later form crusts that subsequently desquamate after one to two weeks. Initial signs and symptoms of MPXV infection are similar to smallpox; however, unlike smallpox, lymphadenopathy is a prominent feature with tender maxillary, cervical and inguinal lymphadenopathy (1-4 cm), seen in 84% of unvaccinated patients, and 54% of vaccinated patients [8,15]. The presence of lymphadenopathy shows that there may be a more robust immune response and recognition of MPXV than variola [15]. Clinical outcomes are usually worse in patients with immunocompromised states, longer extents of exposure to viral particles, and the presence of complications such as bronchopneumonia, encephalitis, and visual loss due to corneal infection [8]. Other complications include hypo-hyperpigmentation, scarring, dehydration (from nausea and vomiting), and secondary bacterial infection leading to septicemia.

Diagnosis

Monkeypox can be suspected in an individual with the aforementioned symptoms, especially if there is a history of contact or travel to areas endemic to monkeypox [18]. A suspected case of monkeypox can be confirmed using the polymerase chain reaction (PCR) test* *[15].

Testing Algorithm

PCR is the gold standard for diagnosis and should be done first. After a negative PCR test, if monkeypox infection is still suspected, other tests listed in Table 1 can be done [15]. After a positive monkeypox PCR test, contact tracing, testing, and, if possible, vaccination of individuals exposed to the patient should be done [6,19].

**Table 1:** Diagnostic tests for monkeypox PCR, polymerase chain reaction; NAAT, nucleic acid amplification test; DNA, deoxyribonucleic acid; IgG, immunoglobulin G; IgM, immunoglobulin M

Tests	Description	Sample used
PCR	It is based on NAAT; for the detection of monkeypox DNA, real-time PCR is currently the gold standard.	Lesion fluid
Viral culture	The virus is grown and isolated from a patient sample.	Lesion fluid
Electron microscopy	An electron microscope is used to morphologically identify pox viruses.	Biopsy specimen, scab material, vesicular fluid
Immunohistochemistry	Tests are conducted for the presence of *Orthopoxvirus*-specific antigens.	Biopsy specimen
Anti-Orthopoxvirus IgG and IgM tests	These tests can be used to assess a recent or remote exposure to *Orthopoxvirus*.	Blood specimen

Management

At the moment, there are no specific treatments approved for monkeypox. Fortunately, the clinical course of monkeypox infection is usually mild and self-limiting. Therefore, it seldom warrants specific therapy, and treatment is often supportive [6,9]. Supportive therapy may include antipyretics for fever, analgesics for pain, or antibiotics for secondary bacterial infections. However, certain patients may require specific treatment. Those with severe disease, immunocompromised patients, pregnant women, and the pediatric age group may require specific treatment [6]. Due to the similarities MPXV shares with smallpox, drugs and vaccines initially intended to treat smallpox have shown signs of efficacy against MPXV. However, limited data is available to support this [2]. Tecovirimat is an antiviral medication approved by the United States Food and Drug Administration (FDA) and the European Medicines Agency (EMA) for the treatment of human smallpox disease [6]. Tecovirimat is available in oral (200 mg capsule) and intravenous formulations. According to the Centers for Disease Control and Prevention (CDC), it can be used as a treatment for MPXV in the United States [6]. Cidofovir or brincidofovir can also be used; these are the antiviral medications that the FDA approves for treating cytomegalovirus (CMV) and human smallpox disease, respectively [6]. Vaccinia Immune Globulin Intravenous (VIGIV) is an immunoglobulin used to treat complications from vaccinia vaccination. The US CDC allows its use as a treatment for monkeypox disease under an expanded access protocol [6].

Prevention

Studies have shown that smallpox vaccination effectively prevents other *Orthopoxvirus* infections, including monkeypox. When administered early in the incubation period, it can prevent disease onset or mitigate the severity of the illness. However, there is a risk of severe adverse effects in immunocompromised patients. The eradication of smallpox in 1980 led to the termination of vaccination efforts against the viral illness, leaving many people susceptible to monkeypox. Next-generation smallpox vaccines, which include ACAM2000 (live vaccinia virus), Modified Vaccinia Ankara (attenuated vaccinia virus), and LC16m8 (attenuated vaccinia virus), with attenuated strains not only offer an improved safety profile compared to first- and second-generation smallpox vaccines but also adequately stimulate antibody production in atopic and immunocompromised patients [9].

Hospitalized patients should be under strict airborne isolation in a negative pressure room. Health personnel should wear appropriately fitted N95 masks, gloves, and eye protection before coming in contact with these patients until the lesions have crusted and scabs have fallen off [16].

## Conclusions

It is pertinent to note that many countries with confirmed monkeypox cases in the May 2022 outbreak are not endemic to monkeypox, and the cases had no travel links to endemic areas. Nevertheless, monkeypox's public health implications and pandemic potential must be studied extensively in light of the devastation caused by the COVID-19 pandemic. A better understanding of factors affecting the spread of this virus will aid in ensuring public health preparedness and developing strategies against future threats. The possibility of underreporting must also be considered as most cases are in rural Africa, countries plagued by poor health infrastructure and scarce resources.
